# The obstinate question of vein graft versus arterial conduits

**DOI:** 10.1093/icvts/ivad131

**Published:** 2023-10-06

**Authors:** Rahul Bhushan, Vijay Grover

**Affiliations:** AlIMS, Patna, New Delhi, India; ABVIMS and Dr RML Hospital, New Delhi, India

**Keywords:** Coronary, Saphenous vein graft, Graft patency

Dear Editor,

We read the article by Janiec *et al.* [1] ‘Symptomatic late saphenous vein graft failure in coronary artery bypass surgery’ with profound interest. The article offers a robust data analysis in evaluation of late saphenous vein graft failure over 23 years and its contribution to symptom recurrence in postcoronary bypass surgery patients among the Swedish population.

However, there are multiple factors that need to be addressed in a given study. First, the authors have included an age group of 40–80 years. The use of venous graft in the younger subgroup below 55 years of age goes against the recommendation of AHA supporting arterial conduits [2]. Similarly, based on data interpretation, roughly 27 000 patients (37.49%) among a total of 71 919 study participants underwent only LIMA to LAD anastomosis. Surgical revascularization with only a single graft seems debateable and adds a bias to selection methods.

Second, the absence of data availability on sequential grafting impacts the inference of the study as much longer patency with better distal run-offs is expected in this subgroup and is globally preferred backed by evidence [3]. There is a lack of data on the degree of vessel collateralization, Syntax score, functional flow rate evaluation and left ventricular function assessment, which plays a pivotal role in determining graft patency.

With the primary end point of study being the recurrence of angina/infarction postoperatively or death, a wide pool of patients with graft failure but being asymptomatic (with multiple distal grafts and adequate collateralization) gets missed. A better follow-up could have been a routine screening postoperative angiogram at fixed time interval for the whole group.

Roughly 20% of LIMA graft failure was reported in 1 year of follow-up in a given study which is surprisingly high and almost twice in comparison to other study groups [4]. Harvesting methods and suture practices thus need an introspect. Nevertheless, it is a fascinating and laudable effort to put forth into discussion a nagging question up for discussion on conduits of choice in surgical revascularization.
